# Soluble Interleukin-2 Receptor: A Potential Marker for Monitoring Disease Activity in IgG4-Related Disease

**DOI:** 10.1155/2018/6103064

**Published:** 2018-03-01

**Authors:** A. F. Karim, L. E. M. Eurelings, R. D. Bansie, P. M. van Hagen, J. A. M. van Laar, W. A. Dik

**Affiliations:** ^1^Section Clinical Immunology, Department of Internal Medicine, Erasmus University Medical Center, Rotterdam, Netherlands; ^2^Section Clinical Immunology, Department of Immunology, Erasmus University Medical Center, Rotterdam, Netherlands; ^3^Laboratory Medical Immunology, Department of Immunology, Erasmus University Medical Center, Rotterdam, Netherlands

## Abstract

**Background:**

IgG4-related disease (IgG4-RD) is a fibroinflammatory condition. T-cells play a crucial role in the pathogenesis, and therefore, serum soluble interleukin-2 receptor (sIL-2R) may be a potential biomarker.

**Method:**

We studied the levels of sIL-2R in 26 histologically proven IgG4-RD patients with available serum sIL-2R and compared them to those in newly diagnosed and untreated sarcoidosis patients (*n* = 78) and controls (*n* = 101) and the serum sIL-2R levels in patients after treatment of IgG4-RD (*n* = 15). The disease activity was measured using the IgG4-Related Disease Responder Index (IgG4-RD RI).

**Results:**

Median serum sIL-2R in IgG4-RD patients was 4667 pg/ml compared to 1515 pg/ml in controls (*P* < 0.001) and 6050 pg/ml in sarcoidosis patients (*P* = 0.004 compared to IgG4-RD). All IgG4-RD patients had elevated serum sIL-2R levels compared to the reference value of <2500 pg/ml in controls and 85% elevated serum IgG4; however, these did not correlate with each other. Both serum sIL-2R and IgG4 levels declined significantly after treatment (*P* = 0.001 and *P* = 0.01, resp.). Before treatment, serum sIL-2R level and IgG4-RD RI did not correlate with each other. However, the decrease in serum sIL-2R upon treatment did correlate significantly (*P* = 0.04) with the decrease in disease activity assessed by IgG-RD RI.

**Conclusion:**

Serum sIL-2R is elevated in IgG4-RD reflecting the inflammatory process with enhanced T-cell activation. Furthermore, serum sIL-2R might serve as a potential marker of response to treatment in IgG4-RD.

## 1. Introduction

IgG4-related disease (IgG4-RD) is a systemic fibroinflammatory condition characterized by storiform fibrotic lesions and accumulation of IgG4-producing plasma cells in the affected tissues [1–3]. It may mimic infectious, chronic inflammatory, and malignant disorders causing delay in diagnosis [2, 3]. Yet, early diagnosis and treatment is important to avoid irreversible organ damage due to fibrosis or secondary amyloidosis in cases of longstanding high-inflammatory conditions [4, 5]. Histology remains the gold standard in the diagnosis of IgG4-RD [2]. Serum IgG4 is used in the diagnosis of this disease. However, it is not a sensitive biomarker and may be normal in histology proven cases [2].

The pathogenesis of IgG4-RD is mostly unclear, but B cells, IgG4-positive plasma cells, and IgG4 antibodies as well as the oligoclonal expansion of T-cells seem to play an important role in the immunopathophysiology [6–9]. T follicular helper-2 (Tfh2) cells are involved in driving the class switch to IgG4 [10]. Cytokines, including interleukin- (IL-) 4 and transforming growth factor *β*, derived from T-helper 2 (Th2) cells and regulatory T-cells, may also contribute to the pathophysiology of IgG4-RD [9, 11–13]. However, the exact role of Th2 cells and their specific cytokines in IgG4-RD is still a subject of debate [14, 15]. Also, CD4+ T-cells that display cytotoxic features are abundant in the peripheral blood and diseased tissue sites of IgG4-RD patients and may contribute to the chronic inflammatory/fibrotic network via secretion of specific cytokines [8, 16].

Peripheral blood levels of soluble IL-2 receptor (sIL-2R) reflect the level of T-cell activation. Elevated serum levels of sIL-2R correlate with disease activity in rheumatoid arthritis and sarcoidosis, diseases in which enhanced T-cell activity is centrally involved [17–20]. Moreover, increasing serum sIL-2R levels may precede T-cell-driven fibrotic responses [21]. In light of the above and as suggested previously [22], serum sIL-2R may also be elevated in IgG4-RD and represent a maker for monitoring disease activity.

Here we examined sIL-2R serum levels in a cohort of histologically diagnosed and therapy-naïve patients with IgG4-RD in comparison to patients with untreated sarcoidosis and controls (blood bank donors). Moreover, serum sIL-2R levels were related to clinical response upon treatment.

## 2. Methods

### 2.1. Study Population

The Erasmus MC University Medical Center represents a national referral center for patients with IgG4-RD. Medical records of patients with IgG4-RD between 1999 and July 2017 were reviewed for clinical characteristics and the availability of serum sIL-2R measurements. Only patients with histologically confirmed IgG4-RD according to established Boston criteria for histology [23], and of whom serum sIL-2R levels were available, were included. In total, serum sIL-2R levels were available from 26 patients with clinically active IgG4-RD. The serum sIL-2R levels from these patients were compared to those of patients with histologically proven sarcoidosis (*n* = 78, from the Erasmus MC University Medical Center sarcoidosis database) and controls (*n* = 101, anonymous blood bank donors). Serum sIL-2R levels in untreated IgG4-RD patients were compared to serum sIL-2R levels after starting treatment (available in 15 patients). The disease activity at the time of measurement of serum sIL-2R levels before and after starting treatment was assessed using the IgG4-RD Responder Index (IgG4-RD RI) [24]. This study was performed according to the Declaration of Helsinki and was approved by the Medical Ethics Committee of Erasmus MC (ethics approval numbers MEC-2014-476, MEC-2015-200, and MEC-2017-084).

### 2.2. Analysis of Serum Parameters

Serum IgG4 and sIL-2R levels were measured by the standard laboratory diagnostic facility within Erasmus MC. IgG4 was measured by immunonephelometry using a Siemens BN II nephelometer, and serum sIL-2R levels were determined with ELISA (Diaclone, Besancon Cedex, France) according to manufacturer instructions in pg/ml. The reference range of sIL-2R is set at <2500 pg/ml within Erasmus MC, based on the serum sIL-2R measurements in 101 blood bank donors. C-reactive protein (CRP) was determined from plasma on a Roche Cobas 8000 analyzer using an immunoturbidimetric assay (Roche Diagnostics). In short, human CRP agglutinates with latex particles coated with monoclonal anti-CRP antibodies. The aggregates are determined turbidimetrically.

### 2.3. Statistical Analysis

Characteristics of the patients with IgG4-RD and sarcoidosis and the controls were described using descriptive statistics. We tested for differences between the three groups of IgG4-RD, sarcoidosis, and controls using a one-way ANOVA. To investigate the individual differences between the groups (IgG4-RD versus controls, sarcoidosis versus controls, and IgG4R versus sarcoidosis), we used independent sample *t*-tests. Because we used multiple *t*-tests, this could be classified as multiple testing. Therefore, we used a stricter *P* value for those 3 specific tests (*P* < 0.01 is considered significant). For all other statistical tests performed, we used the standard *P* value (*P* < 0.05 is considered significant). To investigate whether serum sIL-2R levels changed after treatment, a dependent sample *t*-test was performed. When calculating correlations, we used the Spearman's rank correlation coefficient. The statistical analyses were performed using IBM SPSS statistics 21.0.0 for Windows (SPSS Inc., Chicago, IL, USA).

## 3. Results

### 3.1. Patient Characteristics

Table 1 demonstrates the characteristics of patients with IgG4-RD. Patients with IgG4-RD represent a heterogeneous group, and 58% of the patients presented with involvement of more than one organ.  Table 2 shows the descriptive characteristics of patients with IgG4-RD and sarcoidosis and controls and the serum levels of sIL-2R. There was no difference between the mean age of IgG4-RD patients and sarcoidosis patients (*P* = 0.311). Significantly more patients with IgG4-RD were male (76.9%). The blood bank donors were anonymous. Therefore, no data was present on the age or sex of these volunteers.

### 3.2. sIL-2R Levels in the Study Population

**Figure 1:**
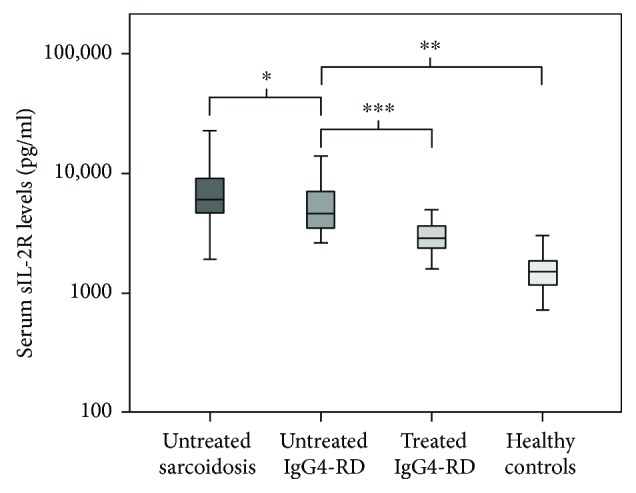
Boxplot of serum sIL-2R levels. Boxplots of serum sIL-2R (pg/ml) in newly untreated sarcoidosis and IgG4-RD and sIL-2R levels after initiation of treatment in IgG4-RD and in control population. ^∗^*P* = 0.004; ^∗∗^*P* < 0.001; ^∗∗∗^*P* = 0.001.

The median level of serum sIL-2R in IgG4-RD was 4667 pg/ml; in sarcoidosis, 6050 pg/ml; and in the control population, 1515 pg/ml. Serum sIL-2R levels in IgG4-RD were significantly higher compared to those in controls (*P* < 0.001) and lower compared to those in sarcoidosis (*P* = 0.004) (Table 2, Figure 1).

### 3.3. sIL-2R Levels after Treatment in IgG4-RD

Serum sIL-2R levels of 15 IgG4-RD patients were also available after treatment and decreased significantly from 5300 pg/ml (3695–6135) to 2864 pg/ml (2160–3653) (*P* = 0.001). The duration of treatment varied per patient, but all patients of whom serum sIL-2R levels were available after treatment showed significant clinical improvement on IgG-RD RI in response to treatment (*P* = 0.01, Table 3).

### 3.4. The Correlation of Serum sIL-2R with Serum IgG4, CRP, and IgG-RD Responder Index

**Figure 2:**
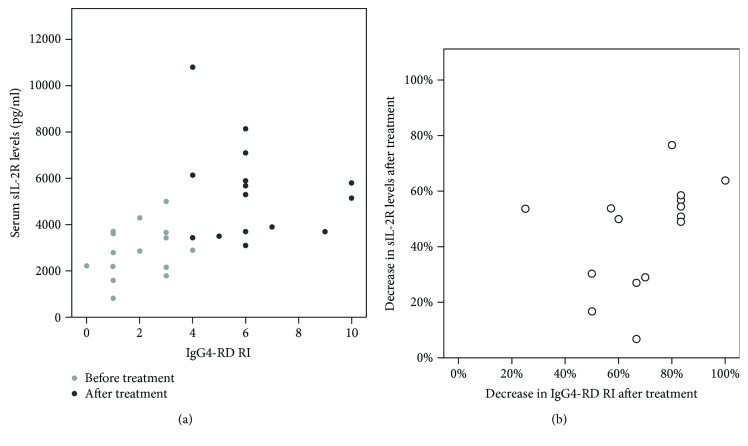
Correlation between sIL-2R and IgG4-RD RI. Correlation between sIL-2R and IgG4-RD RI before and after treatment (a) and correlation between the decrease in IgG4-RD RI after treatment and the decrease in sIL-2R levels after treatment (b).

In 19 patients with IgG4-RD, serum IgG4 levels were available before and after treatment and decreased significantly (*P* = 0.001) from 4.0 g/l (1.7–10.3) to 1.8 g/l (1.3–3.3) and correlated with a clinical improvement of the disease. In all IgG4-RD patients, serum sIL-2R was elevated, compared to 85% (22/26) for serum IgG4 (Table 1). No significant correlation was observed between the levels of serum sIL-2R and serum IgG4 before (Spearman's rho: −0.08, *P* = 0.776) and after (Spearman's rho: 0.225, *P* = 0.450) treatment. Elevated CRP levels (CRP ≥ 10 mg/l) were found in 27% (7/26) of patients before treatment. No significant correlation was observed between the levels of CRP and serum sIL-2R before (Spearman's rho: 0.299, *P* = 0.279) and after (Spearman's rho: 0.379 *P* = 0.164) treatment. Serum sIL-2R and IgG4-RD RI did not correlate with each other before treatment (Spearman's rho: −0.119, *P* = 0.672). However, the decrease in serum sIL-2R significantly correlated with the decrease in disease activity assessed by IgG-RD RI (Figure 2, Spearman's rho: 0.527, *P* = 0.044).

## 4. Discussion

Here we demonstrate that serum sIL-2R levels are elevated in all patients with active and untreated IgG4-RD. Furthermore, the decrease in sIL-2R levels after treatment significantly correlates with clinical improvement in a small cohort of patients with IgG4-RD.

It has been described that sIL-2R levels may differ with age, with children (age 1–14 years) and elderly (age 67–99 years) having higher sIL-2R levels as compared to (young) adults (age 22–67) [20, 25]. We used anonymous blood donors as the control in our study. In the Netherlands, blood donors will be mostly within this (young) adult age group, as were most of the IgG4-RD patients (and sarcoidosis patients) included in our study. Therefore, we consider it unlikely that age differences between groups influenced the outcome of our study. This is further supported by previous studies that described elevated sIL-2R levels in IgG4-RD, especially in relation to the higher percentage (>40%) of IgG4-positive plasma cells in the salivary gland tissues [22], glycolysis within the tissues [26], and increased levels of CC-chemokine ligand 18 (CCL18), a substantial marker for fibrotic diseases [27].

Our observations in patients with clinically active IgG4-RD suggest that serum sIL-2R reflects the inflammatory process in these patients. sIL-2R is secreted by activated T-cells, and elevated levels are used as marker for T-cell activity in other inflammatory diseases [28]. Although the pathophysiological mechanism of IgG4-RD is not yet fully elucidated, T-cell activation is currently considered an important contributor [6, 8, 9]. The levels of serum sIL-2R and the disease activity score (IgG4-RD RI) before treatment did not show a significant correlation, but our observation of declining sIL-2R serum levels upon clinical improvement after immunosuppressive treatment supports the pathological role of (excessive) T-cell activity in IgG4-RD and further indicates that serum sIL-2R levels reflect IgG4-RD disease activity. Although the posttreatment measurement intervals of serum sIL-2R differed in patients, it will probably not have influenced the results, because the decrease in sIL-2R correlated with clinical improvement of the disease activity obtained by IgG4-RD RI.

Theoretically, serum IgG4 levels might reflect disease activity in IgG4-RD. However, in general, serum IgG4 is normal in ~30% of IgG4-RD patients [1]. Indeed 15% of our patients with IgG4-RD had normal serum IgG4 levels, whilst serum sIL-2R was elevated in all patients. Moreover, there was no direct correlation between serum IgG4 and sIL-2R and between CRP and serum sIL-2R levels in the cohort studied.

Sarcoidosis often presents with clinical presentations similar to IgG4-RD and is associated with elevated serum sIL-2R (this study and others), and if untreated, fibrosis may develop [17, 18, 29]. Consequently, sarcoidosis represents an appropriate disease control for comparison of serum sIL-2R levels with IgG4-RD. The serum levels of sIL-2R were significantly lower in IgG4-RD compared to sarcoidosis, yet significantly higher than that observed in the control population. This may be indicative of a more vigorous T-cell component, or burden of activated T-cells, in sarcoidosis. Because sIL-2R is a non-disease-specific T-cell activation marker, it might not suite as a specific diagnostic tool in IgG4-RD [19]. However, as all IgG4-RD patients displayed elevated serum sIL-2R, compared to 85% and 27% for serum IgG4 and CRP, respectively, its negative predictive value can be considered high and thus may be helpful in diagnostic evaluation of IgG4-RD. Moreover, sIL-2R could be useful in monitoring disease activity, disease dynamics, and early detection of a recurrence.

This study is limited by its retrospective character and the relatively small population. Therefore, larger studies are required to obtain the sensitivity and specificity of sIL-2R in IgG4-RD.

In conclusion, we demonstrate that serum sIL-2R is elevated in IgG4-RD. Furthermore, serum sIL-2R may have potential as a tool for monitoring disease activity/treatment response in IgG4-RD. The value of serum sIL-2R for this application needs further confirmation in prospective and larger studies, also in comparison to diseases with the capacity to mimic IgG4-RD such as granulomatosis with polyangiitis.

## Figures and Tables

**Table 1:** Characteristics of patients with IgG4-RD.

*P*	Sex	Age	Disease manifestation of IgG4-RD	IgG4 before treatment	sIL-2R before treatment	Treatment/comments
1	M	53	Orbit and lymph nodes	17.7	3695	Rx: prednisone
2	M	36	Salivary gland	3.18	3441	Rx: initially prednisone and Aza, later RTX
3	M	42	Lymph nodes and kidney	3.0	12,400	Rx: prednisone and RTX, now CellCept
4	M	60	Orbit and lymph nodes	5.22	3700	Rx: prednisone and MTX
5	F	46	Orbit	2.76	3400	Rx: now infliximab
6	M	61	Orbit, lymph node, and prostate	13.48	5800	sIL-2R initially measured under low-dose prednisone but active disease. Now prednisone and MTX
7	M	57	Orbit, lymph node, and prostate and pancreas	13.40	5151	Rx: prednisone
8	M	63	Orbit, pancreas, ENT, and prostate	1.65	5700	Rx: prednisone
9	M	17	Lung, lymph nodes, and brain	10.30	3900	Rx: prednisone and RTX
10	M	53	Mesenteric manifestation	25.25	5900	Rx: prednisone and RTX
11	M	32	Pericardial and pleural manifestation	5.40	3500	Rx: prednisone
12	M	39	Orbit	1.65	8135	Rx: prednisone and Aza
13	M	29	Lymph node (Kimura disease)	1.17	3800	Patient declined treatment
14	M	55	Hypophysis and ENT	1.57	3100	Rx: prednisone
15	M	48	Mesenteric manifestation	3.01	14,027	Patient did not show up for follow-up/treatment
16	F	52	Orbit	0.61	2859	Surgical resection periorbital mass, no systemic treatment
17	M	61	Biliary tract and ENT	5.55	5300	Rx: prednisone
18	M	79	Lymph node	42.8	9000	Only lymph node manifestation, no systemic treatment
19	F	39	Salivary gland	3.97	3374	Rx: prednisone
20	M	74	Orbit and lymph node	8.82	6135	Rx: dexamethasone
21	F	52	Orbit and lymph node	3.27	2639	Rx: prednisone
22	M	74	Orbit and ENT	3.30	7977	Status after ocular surgery, currently no systemic treatment
23	F	61	Orbit and ENT	0.36	4182	Rx: prednisone
24	M	65	Retroperitoneal fibrosis	3.44	7100	Rx: prednisone
25	M	53	Orbit and ENT	1.45	4105	Rx: prednisone, MTX, and now RTX
26	F	59	Skin	1.18	10,800	Rx: prednisone and Plaquenil

Characteristics of patients with IgG4-RD, including serum IgG4 and serum sIL-2R before treatment. P = patient; Rx = treatment; MTX = methotrexate; Aza = azathioprine; RTX = rituximab, NA = not applicable; NM = not measured. Normal range of serum IgG4 is 0.08–1.40 g/l, and normal range of serum sIL-2R is below 2500 pg/ml.

**Table 2:** The descriptive characteristics of the study population.

	IgG4-RD patients	Sarcoidosis patients	Controls (anonymous blood bank donors)
Number	26	78	101
Median age (IQR)	53 years (41–61)	49 years (38–56)	Not applicable
Males	20 (76.9%)	37 (47.7%)	Not applicable
Females	6 (23.1%)	41 (52.6%)	Not applicable
Median serum sIL-2R (IQR)	4667 pg/ml (3485–7319)	6050 pg/ml (4651–9475)	1515 pg/ml (1150–1880)

**Table 3:** Treatment response in IgG4-RD.

P	IgG4 before treatment	IgG4 after treatment	sIL-2R before treatment	sIL-2R after treatment	CRP before treatment	CRP after treatment	IgG4-RD RI before treatment	IgG4-RD RI after treatment
1	17.7	10.10	3695	3443	1.8	1.6	9	3
2	3.18	3.32	3441	2864	1.0	2.5	4	2
4	5.44	0.46	3700	1600	0.5	0.9	6	1
6	13.48	6.70	5800	2900	0.3	0.3	10	4
7	13.40	3.27	5151	3653	4.7	1.4	10	3
8	1.65	0.28	5700	2800	1.0	0.6	6	1
9	10.30	4.99	3900	1800	50.0	4.5	7	3
10	25.25	4.58	5900	4300	80.0	35.0	6	2
11	5.40	2.79	3500	819	4.8	0.4	5	1
12	1.65	1.54	8135	3700	10.0	7.5	6	1
14	1.57	1.33	3100	2160	5.2	10.0	6	3
17	5.55	1.84	5300	2200	0.3	0.3	6	1
20	8.82	1.28	6135	2222	2.8	38	4	0
24	3.44	1.35	7100	3616	31	6.8	6	1
26	1.29	1.76	10,800	5000	8.0	7.6	4	3

Treatment response in IgG4-RD with levels of serum IgG4, CRP, and sIL-2R before and after treatment. The disease activity has been measured using the IgG4-RD RI. Normal range of serum IgG4 is 0.08–1.40 g/l, normal range of serum sIL-2R is below 2500 pg/ml, and normal range of CRP is below 10 mg/l. CRP = C-reactive protein; IgG4-RD RI = IgG4-Related Disease Responder Index.
